# Early Postoperative Biomarkers Associated with Subsequent Operative Source Control in Anastomotic Leakage After Cytoreductive Surgery and Hyperthermic Intraperitoneal Chemotherapy: A Multicenter Retrospective Cohort Study

**DOI:** 10.3390/diagnostics16162647

**Published:** 2026-08-19

**Authors:** Erkan Güler, Kamil Erözkan, Volkan Sayur, Sami Benli, Cumhur Özcan, Osman Bozbıyık, Cemil Çalışkan, Hamza Çınar, Tufan Gümüş, Gülşah Selvi Demirtaş, Tahsin Çolak, Taylan Özgür Sezer

**Affiliations:** 1 Department of Surgery, Division of Surgical Oncology, Mersin University School of Medicine, Mersin 33110, Türkiye; benlisami@outlook.com (S.B.);; 2 Department of Surgery, Ege University School of Medicine, İzmir 35100, Türkiyesayurvolkan@gmail.com (V.S.); bozbiyiko@gmail.com (O.B.); cemil.caliskan@ege.edu.tr (C.Ç.); dr.tufan.gumus@gmail.com (T.G.);; 3 Department of Surgery, Mersin University School of Medicine, Mersin 33110, Türkiye; cumhurozcan79@gmail.com; 4 Department of Surgery, Division of Gastrointestinal Surgery, Pamukkale University School of Medicine, Denizli 20010, Türkiye; drhamzacinar@gmail.com; 5 Department of Obstetrics and Gynecology, Division of Gynecologic Oncology, Denizli State Hospital, Denizli 20010, Türkiye; drg.selvi@gmail.com

**Keywords:** anastomotic leakage, CRS-HIPEC, C-reactive protein, neutrophil-to-lymphocyte ratio, operative source control

## Abstract

**Background/Objectives:** Anastomotic leakage is a serious complication after cytoreductive surgery with hyperthermic intraperitoneal chemotherapy (CRS-HIPEC). Among patients who develop leakage, early identification of those requiring operative source control remains difficult. This study evaluated the discriminatory performance of early postoperative inflammatory and metabolic biomarkers for subsequent operative source control. **Methods:** This multicenter retrospective cohort study screened 540 patients who underwent CRS-HIPEC between January 2015 and January 2025. Anastomotic leakage was identified in 51 patients. Four patients underwent reoperation before completion of the postoperative day (POD) 3 laboratory assessment: two for anastomotic leakage and two for postoperative bleeding. Three additional patients were excluded because the clinical or laboratory data required for the primary analyses were unavailable. The final cohort comprised 44 patients, of whom 24 (54.5%) subsequently underwent operative source control for anastomotic leakage and 20 (45.5%) were managed without reoperation through one month. C-reactive protein (CRP), albumin, neutrophil-to-lymphocyte ratio (NLR), white blood cell count, glucose, CRP-to-albumin ratio (CAR), and leukoglycemic index (LGI) were assessed on POD1 and POD3. All POD1 and POD3 laboratory measurements were obtained before operative source control in patients who subsequently underwent reoperation. Discriminatory performance was evaluated using receiver operating characteristic analysis. Post hoc, data-informed Firth penalized logistic regression models were used to explore the associations of POD1 CRP and POD3 NLR with operative source control after adjustment for BMI and HIPEC agent. **Results:** NLR was significantly higher in patients who subsequently required operative source control on both POD1 (median, 14.9 vs. 9.8; *p* < 0.001) and POD3 (21.0 vs. 9.8; *p* < 0.001). The increase in NLR from POD1 to POD3 was also greater in the operative group (median change, 4.8 vs. 1.0; *p* = 0.009). POD3 NLR showed the highest observed discriminatory performance (area under the curve [AUC], 0.901; 95% confidence interval [CI], 0.810–0.992), while POD1 NLR (AUC, 0.834; 95% CI, 0.717–0.951) and POD1 CRP (AUC, 0.798; 95% CI, 0.649–0.947) also showed good observed discrimination. POD3 CRP-to-albumin ratio showed moderate discrimination, whereas POD3 LGI showed more limited performance. In post hoc exploratory Firth models adjusted for BMI and HIPEC agent, POD1 CRP per 10 mg/L increase (adjusted odds ratio [aOR], 1.52; 95% CI, 1.10–2.56; *p* = 0.006) and POD3 NLR per 5-unit increase (aOR, 3.91; 95% CI, 1.57–20.23; *p* = 0.001) remained associated with operative source control. All ROC-derived cutoff values and their operating characteristics were data-derived, intended solely for hypothesis generation, and should not be used for clinical decision-making. **Conclusions:** Within this selected retrospective cohort, POD3 NLR showed the highest observed discriminatory performance and remained associated with subsequent operative source control in a post hoc exploratory adjusted model. POD1 NLR and POD1 CRP also provided exploratory discriminatory information, while the greater increase in NLR from POD1 to POD3 suggests that serial biomarker trajectories warrant further investigation. These findings, including the data-derived cutoff estimates, are intended solely for hypothesis generation and should not be used for clinical decision-making. Prospective external validation is required.

## 1. Introduction

Cytoreductive surgery combined with hyperthermic intraperitoneal chemotherapy (CRS-HIPEC) is an established treatment strategy for selected patients with peritoneal surface malignancies. Achieving complete or near-complete cytoreduction frequently requires extensive peritonectomy procedures, multivisceral resections, and the construction of one or more gastrointestinal anastomoses [1]. Anastomotic leakage remains one of the most serious complications following CRS-HIPEC and has been reported in approximately 5–10% of patients in recent series [2,3,4]. In addition to increasing postoperative morbidity and mortality, leakage may lead to prolonged hospitalization, repeated interventions, stoma formation, and delays in subsequent oncological treatment.

Once an anastomotic leak has developed, the clinical course and required treatment may vary considerably. Clinically stable patients with localized contamination may be successfully managed with antimicrobial treatment, image-guided drainage, or endoscopic interventions, whereas patients with uncontrolled contamination, generalized peritonitis, clinical deterioration, or failure of conservative management require operative source control [5]. The decision to proceed with reoperation is therefore based on a combination of physiological status, radiological findings, the extent of contamination, and response to initial treatment. However, these findings may evolve gradually, and delayed recognition of inadequate source control may adversely affect outcomes. Identifying objective laboratory parameters that distinguish patients likely to require operative source control at an early stage could therefore support clinical decision-making and closer surveillance.

Postoperative inflammatory biomarkers have been extensively investigated for the early detection of complications after major abdominal surgery. In patients undergoing CRS-HIPEC, elevated C-reactive protein (CRP) concentrations, particularly from postoperative day (POD) 3 onward, have been associated with major and infectious postoperative complications [6,7]. Evidence from colorectal surgery has similarly demonstrated that postoperative CRP has useful discriminatory and rule-out performance for anastomotic leakage, while the neutrophil-to-lymphocyte ratio (NLR) may provide complementary information regarding the systemic inflammatory response [8,9,10,11]. Composite markers may capture additional physiological dimensions: the CRP-to-albumin ratio combines systemic inflammation with nutritional and metabolic reserve, whereas the leukoglycemic index (LGI) integrates leukocytosis and stress hyperglycemia. Although LGI has demonstrated prognostic value in other postoperative populations, its role following gastrointestinal surgery and CRS-HIPEC remains largely unexplored [12].

Previous studies have primarily evaluated biomarkers for detecting the occurrence of anastomotic leakage or predicting general postoperative complications in unselected surgical populations. Limited evidence is available regarding whether early postoperative inflammatory and metabolic parameters can differentiate, among patients who subsequently develop an anastomotic leak following CRS-HIPEC, those who require operative source control from those managed without reoperation through one month. The present study therefore aimed to evaluate the discriminatory performance of POD1 and POD3 CRP, CRP-to-albumin ratio, NLR, white blood cell count, glucose, and LGI for subsequent operative source control. Changes in these parameters between POD1 and POD3 were also assessed, with an additional exploratory analysis of LGI parameters in patients without diabetes mellitus.

## 2. Materials and Methods

### 2.1. Study Design and Ethical Approval

This multicenter retrospective cohort study included patients treated at three referral centers and five additional patients operated on by the same participating surgeon at multiple private hospitals between January 2015 and January 2025.

The study protocol was approved by the Mersin University Clinical Research Ethics Committee on 23 July 2025 (decision no. 2025/841). The study was conducted in accordance with the principles of the Declaration of Helsinki. Because the study was based on retrospectively collected and anonymized medical-record data, the requirement for individual informed consent was waived by the ethics committee.

### 2.2. Patient Selection

The medical records of 540 patients who underwent CRS-HIPEC during the study period were screened. Patients were eligible if they underwent CRS-HIPEC with a single colonic, enterocolic, colorectal, or enterorectal anastomosis and subsequently developed an anastomotic leak. Patients with more than one gastrointestinal anastomosis and those with an isolated enteroenteric or gastroesophageal anastomosis were excluded during the initial screening. Ileocolic and ileotransverse anastomoses were categorized as enterocolic anastomoses.

Anastomotic leakage was identified in 51 patients. Four patients underwent reoperation before completion of the POD3 laboratory assessment: two for anastomotic leakage and two for postoperative bleeding. Three additional patients were excluded because the clinical or laboratory data required for the primary analyses were unavailable. The final cohort comprised 44 patients, of whom 24 (54.5%) subsequently underwent operative source control for anastomotic leakage and 20 (45.5%) were managed without reoperation through one month.

No a priori sample size calculation was performed because of the retrospective design, the relative rarity of anastomotic leakage following CRS-HIPEC, and the inclusion of all eligible patients available during the study period.

### 2.3. Definition of Anastomotic Leakage

Anastomotic leakage was defined as a clinically relevant defect of the intestinal anastomosis resulting in communication between the intra- and extraluminal compartments. The diagnosis was established using documented clinical, radiological, drain-related, endoscopic, or operative findings. Radiological evidence included extraluminal contrast leakage, a perianastomotic fluid or gas-containing collection, or visible disruption of anastomotic continuity on computed tomography. Clinical and drain-related findings included otherwise unexplained postoperative clinical deterioration, abdominal pain or tenderness, peritoneal signs, fever, sepsis, or unequivocal enteric, bilious, or feculent drainage.

Direct visualization of an anastomotic defect during endoscopy or reoperation was considered definitive evidence of leakage. Drain-fluid biochemical abnormalities were considered supportive evidence only when concordant with the clinical or radiological findings and were not accepted as the sole diagnostic criterion.

### 2.4. Study Groups and Primary Outcome

The primary outcome was subsequent operative source control following the diagnosis of anastomotic leakage. Patients were classified according to their definitive leak-management strategy. The operative group included patients who underwent reoperation for anastomotic leakage. Operative source control procedures included surgical drainage, abdominal lavage, anastomotic revision or takedown, and/or fecal diversion with stoma formation. The non-operative group included patients managed without reoperation through one month using clinical observation, antimicrobial treatment, image-guided percutaneous drainage, and/or endoscopic intervention.

Patients who initially underwent non-operative management but subsequently required reoperation were classified in the operative group. All laboratory measurements included in the discrimination analyses were obtained before operative source control. Management without reoperation through one month was defined as discharge without reoperation during the index hospitalization and the absence of an indication for reoperation at the one-month follow-up visit. Management decisions were not governed by a prospectively standardized protocol across participating centers. Operative or non-operative management was determined by the treating surgical team based on the available clinical and physiological findings, radiological features, drain characteristics, response to initial treatment, and the availability of radiological or endoscopic interventions. However, the exact patient-level indications and relative contribution of each factor were not consistently documented in the pooled retrospective dataset.

### 2.5. Data Collection

Demographic, clinical, operative, and laboratory data were collected retrospectively from institutional medical records and databases. The evaluated baseline and perioperative variables included age, sex, body mass index, diabetes mellitus, receipt of neoadjuvant treatment, peritoneal cancer index, completeness of cytoreduction score, operative duration, HIPEC agent, HIPEC duration, and anastomosis type. HIPEC agents were categorized as oxaliplatin, mitomycin C, or cisplatin. Anastomoses were categorized as colocolic, enterocolic, colorectal, or enterorectal. Ileocolic and ileotransverse anastomoses were included in the enterocolic category. Completeness of cytoreduction was classified as CC-0 or CC-1. Protective stoma status was recorded but was not included in the comparative tables because none of the patients had a protective stoma at the index operation. The baseline and operative variables included in the analysis are presented in Table 1.

### 2.6. Postoperative Laboratory Parameters

The primary laboratory analyses were based on measurements obtained on postoperative days 1 and 3. These time points were selected to evaluate early inflammatory and metabolic changes before operative source control. The evaluated laboratory parameters included C-reactive protein, serum albumin, absolute neutrophil and lymphocyte counts, white blood cell count, and serum glucose. Measurements recorded in the original database as postoperative day 0 corresponded to the first postoperative day and were therefore analyzed and reported as postoperative day 1 values. Postoperative day 5 and day 7 measurements were not included in the primary discrimination analyses because the temporal relationship between these measurements and operative source control could not be established consistently for every patient. Some later measurements may therefore have reflected the biological response after reoperation. Accordingly, the primary analyses were restricted to patients who had not undergone reoperation before completion of the POD3 laboratory assessment. The postoperative timing of anastomotic leakage diagnosis was additionally categorized as POD3–4, POD5–7, POD8–10, or after POD10. Two patients diagnosed with anastomotic leakage on POD1–2 underwent early reoperation and were excluded from the primary cohort. All operative source control procedures in the primary cohort were performed after completion of the POD3 laboratory assessment. Laboratory samples were routinely collected in the morning on POD1 and POD3; however, biomarker assay platforms, postoperative surveillance and imaging practices, and criteria for reoperation were not governed by a prospectively standardized protocol across participating institutions.

### 2.7. Derived Inflammatory and Metabolic Indices

The neutrophil-to-lymphocyte ratio was calculated by dividing the absolute neutrophil count by the absolute lymphocyte count:

NLR = absolute neutrophil count/absolute lymphocyte count

The C-reactive protein-to-albumin ratio was calculated as follows:

CRP-to-albumin ratio = CRP (mg/L)/albumin (g/L)

The leukoglycemic index was calculated as follows:

LGI = white blood cell count (×10^3^/µL) × serum glucose (mg/dL)

Absolute changes in laboratory parameters between postoperative days 1 and 3 were calculated as follows:

Δ parameter = POD3 value − POD1 value

The relative change in the leukoglycemic index was expressed as follows:

POD3/POD1 LGI ratio = POD3 LGI/POD1 LGI

Postoperative day 1 and postoperative day 3 biomarker values and their absolute changes were compared between the operative and non-operative groups.

### 2.8. Diabetes Mellitus Subgroup Analysis

Because serum glucose concentrations and the leukoglycemic index may be influenced by pre-existing diabetes mellitus, a prespecified subgroup analysis was performed among patients without diabetes mellitus. Postoperative day 1 LGI, postoperative day 3 LGI, the absolute change in LGI, and the postoperative day 3-to-day 1 LGI ratio were compared between the operative and non-operative groups in patients without diabetes mellitus.

A separate analysis of patients with diabetes mellitus was not performed because only nine patients had diabetes, which was considered insufficient for reliable diabetes-specific estimates or cutoff values. The non-diabetic subgroup consisted of 35 patients, including 17 patients managed non-operatively and 18 patients who required operative source control.

### 2.9. Statistical Analysis

Continuous variables were summarized as median and interquartile range because of the limited sample size and the non-normal distribution of several variables. Categorical variables were presented as number and percentage. Continuous variables were compared between the operative and non-operative groups using the Mann–Whitney U test. Binary categorical variables were compared using Fisher’s exact test. Categorical variables with more than two levels were evaluated using the Fisher–Freeman–Halton exact test with Monte Carlo simulation based on 300,000 resamples. The discriminatory performance of postoperative biomarkers for subsequent operative source control was assessed using receiver operating characteristic analysis. The area under the receiver operating characteristic curve was reported with its 95% confidence interval. The 95% confidence intervals for the AUCs were calculated using the DeLong method. Statistical significance was assessed by testing the area under the curve against the null value of 0.50. Two-sided 95% confidence intervals for sensitivity, specificity, positive predictive value, and negative predictive value were calculated using the exact binomial Clopper–Pearson method.

Optimal cutoff values were selected using the maximum Youden index. Sensitivity, specificity, positive predictive value, and negative predictive value were calculated at the corresponding cutoff values.

Because the cutoff values were derived and evaluated in the same cohort, they were regarded as exploratory and were not interpreted as externally validated clinical thresholds. A combined receiver operating characteristic figure was generated for four selected biomarkers: postoperative day 1 CRP, postoperative day 3 NLR, postoperative day 3 CRP-to-albumin ratio, and postoperative day 3 LGI. These biomarkers were selected to represent early inflammatory response, postoperative day 3 inflammatory response, a composite inflammatory–nutritional index, and a combined inflammatory–metabolic index, respectively. Receiver operating characteristic analyses were also performed for LGI parameters in patients without diabetes mellitus. These analyses included postoperative day 1 LGI, postoperative day 3 LGI, the absolute change in LGI, and the postoperative day 3-to-day 1 LGI ratio.

As a post hoc, data-informed adjusted analysis, separate Firth penalized logistic regression models were constructed for POD1 CRP and POD3 NLR, which were selected after review of their observed discriminatory performance and clinical relevance, with subsequent operative source control as the dependent variable. Each model was adjusted for BMI and HIPEC agent, the two significantly imbalanced covariates with complete data. POD1 CRP and POD3 NLR were scaled per 10 mg/L and per 5-unit increase, respectively. Mitomycin C was used as the reference category for HIPEC agent. Neoadjuvant treatment was not included because data were unavailable for nine patients, and complete-case adjustment would further reduce the already limited sample size and increase model instability. Adjusted odds ratios were reported with profile-likelihood 95% confidence intervals and penalized likelihood-ratio *p* values. These adjusted models were exploratory, were not prespecified, and were not intended as confirmatory analyses.

Missing data were not imputed. Neoadjuvant treatment data were unavailable in the source medical records for nine patients (three non-operative and six operative), possibly because some patients had received oncological treatment at outside institutions. Analyses involving variables with missing values were performed using available-case analysis, and the number of available observations was reported where applicable. No adjustment for multiple comparisons was performed because the analyses were exploratory.

Descriptive analyses and receiver operating characteristic curves were performed using jamovi version 2.7.28. Firth penalized logistic regression analyses were performed using a custom Python implementation based on SciPy version 1.17.0 (Python version 3.13.5).

### 2.10. Center-Level Effects

Institution-level patient and operative source control counts were reported descriptively for the three referral centers. Five additional patients were treated by the same participating surgeon at multiple private hospitals and therefore could not be assigned to a single institutional center. Owing to the small number of patients within each institution and the non-uniform institutional origin of the private-hospital cases, center-level clustering or adjusted comparisons were not performed.

## 3. Results

### 3.1. Study Population and Baseline Characteristics

Among 540 patients who underwent CRS-HIPEC between 2015 and 2025, anastomotic leakage was identified in 51 patients. Seven patients were excluded: two underwent reoperation for anastomotic leakage before completion of the POD3 laboratory assessment, two underwent early reoperation for postoperative bleeding, and three had unavailable clinical or laboratory data required for the primary analyses. The final cohort comprised 44 patients; 24 patients (54.5%) underwent subsequent operative source control, whereas 20 patients (45.5%) managed without reoperation through one month. Ege University contributed 18 patients, of whom 10 underwent operative source control; Mersin University contributed 14 patients, of whom 7 underwent operative source control; Denizli State Hospital contributed 7 patients, of whom 4 underwent operative source control; and an additional five patients were operated on by the same participating surgeon across multiple private hospitals, three of whom underwent operative source control. Among the 44 patients included in the primary cohort, anastomotic leakage was diagnosed on POD3–4 in 7 patients (15.9%), POD5–7 in 17 (38.6%), POD8–10 in 13 (29.5%), and after POD10 in 7 (15.9%). Thus, 37 patients (84.1%) were diagnosed on POD5 or later. The two patients diagnosed on POD1–2 underwent early reoperation and were excluded from the primary analysis. All operative source control procedures in the analyzed cohort were performed after completion of the POD3 laboratory assessment. The median age of the overall cohort was 65 years (IQR, 54–68.2), and 27 patients (61.4%) were female. The median BMI was 26 kg/m^2^ (IQR, 24–32.2), and nine patients (20.5%) had diabetes mellitus. Among the 35 patients with available neoadjuvant treatment data, 17 (48.6%) had received neoadjuvant therapy. The median peritoneal cancer index was 14 (IQR, 9–18), and CC-1 cytoreduction was recorded in 11 patients (25.0%).

Patients who required operative source control had a significantly higher BMI than those managed without reoperation (29 [IQR, 26–35.1] vs. 24 [IQR, 20–26] kg/m^2^; *p* < 0.001). Neoadjuvant treatment was less frequent in the operative group than in the non-operative group (27.8% vs. 70.6%; *p* = 0.018). The distribution of HIPEC agents also differed significantly between groups (*p* = 0.002). Oxaliplatin was administered to 75.0% of patients in the operative group compared with 30.0% in the non-operative group, whereas mitomycin C was used in 25.0% and 50.0%, respectively. Cisplatin was used in four patients in the non-operative group and in none of the patients requiring operative source control.

Age, sex, diabetes mellitus, peritoneal cancer index, completeness of cytoreduction, operative duration, and HIPEC duration were comparable between groups. Anastomosis type also did not differ significantly (*p* = 0.095), although enterorectal anastomoses were observed only in the operative group. No patient had a protective stoma at the index operation (Table 1).

Among the 24 patients who underwent operative source control, all had feculent drain output. Sixteen patients (66.7%) had free leakage with anastomotic dehiscence involving more than one-third of the anastomotic circumference, whereas eight (33.3%) had contained leakage without major anastomotic dehiscence. Among the 20 patients managed without reoperation through one month, 10 (50.0%) had contained leakage and 10 (50.0%) had free leakage. Before reoperation, five patients (20.8%) underwent an endoscopic intervention and one (4.2%) underwent percutaneous drainage. In the non-operative group, 13 of 20 patients (65.0%) underwent percutaneous drainage, none underwent endoscopic intervention, and seven (35.0%) were managed without an additional radiological or endoscopic procedure. Operative source control included surgical drainage or lavage, anastomotic revision or takedown, and/or fecal diversion; however, standardized patient-level indications for reoperation and the exact distribution of individual operative procedures were not consistently available in the pooled dataset. According to the management-based ISREC classification, all 24 patients who underwent reoperation corresponded to Grade C leakage, and the 13 non-operatively managed patients who underwent percutaneous drainage corresponded to Grade B. For the remaining seven patients managed without a radiological or endoscopic intervention, the available retrospective data did not permit a reliable distinction between Grade A and Grade B because patient-level changes in medical treatment were not consistently documented.

### 3.2. Postoperative Inflammatory and Metabolic Parameters

On postoperative day 1, patients who subsequently required operative source control had significantly higher CRP levels than patients managed without reoperation (71 [IQR, 57.8–127.5] vs. 26.5 [IQR, 18–89] mg/L; *p* < 0.001). The POD1 CRP-to-albumin ratio was also higher in the operative group (2.26 [IQR, 1.80–6.07] vs. 1.24 [IQR, 0.65–2.78]; *p* = 0.001). Similarly, POD1 NLR was significantly higher in patients who subsequently required operative source control (14.9 [IQR, 12.4–16.7] vs. 9.8 [IQR, 7.0–12.7]; *p* < 0.001). In contrast, POD1 albumin, WBC count, serum glucose, and LGI did not differ significantly between groups.

By postoperative day 3, the between-group differences were more pronounced. Patients requiring operative source control had higher CRP levels (203 [IQR, 193.8–313] vs. 170 [IQR, 52.5–245.5] mg/L; *p* = 0.004) and higher CRP-to-albumin ratios (7.92 [IQR, 7.25–15.57] vs. 5.31 [IQR, 2.19–10.23]; *p* = 0.003). POD3 NLR showed marked between-group separation, with a median of 21.0 (IQR, 15.6–23.3) in the operative group and 9.8 (IQR, 8.5–12.7) in the non-operative group (*p* < 0.001).

POD3 WBC count was also higher in patients requiring operative source control (17 [IQR, 16–17.2] vs. 14.5 [IQR, 13–17] ×10^3^/µL; *p* = 0.016). Similar differences were observed for POD3 glucose (209 [IQR, 166.5–273.2] vs. 175 [IQR, 146.8–206.5] mg/dL; *p* = 0.046) and POD3 LGI (3060 [IQR, 2800–4722] vs. 2672 [IQR, 1930–3321]; *p* = 0.030). POD3 albumin levels remained comparable between groups.

Evaluation of changes from POD1 to POD3 showed a greater increase in the CRP-to-albumin ratio in the operative group (6.00 [IQR, 5.42–9.57] vs. 2.31 [IQR, 1.53–8.78]; *p* = 0.014). The increase in NLR was also significantly greater among patients requiring operative source control (4.8 [IQR, 2.4–7.4] vs. 1.0 [IQR, −2.7 to 4.1]; *p* = 0.009). Similarly, the increases in WBC count (2.5 [IQR, 2–4] vs. 0.5 [IQR, −1 to 3.2] ×10^3^/µL; *p* = 0.019) and LGI (1015 [IQR, 763–1584] vs. 381 [IQR, −198 to 1371]; *p* = 0.022) were greater in the operative group. Changes in CRP, albumin, and glucose were not significantly different between groups. The POD3/POD1 LGI ratio was numerically higher in the operative group but did not reach statistical significance (1.44 [IQR, 1.32–1.58] vs. 1.19 [IQR, 0.91–1.48]; *p* = 0.061) (Table 2).

### 3.3. Leukoglycemic Index in Patients Without Diabetes Mellitus

The analysis restricted to patients without diabetes mellitus included 35 patients, of whom 18 required operative source control and 17 were managed without reoperation. POD1 LGI was similar between the operative and non-operative groups (2074 [IQR, 2032–3056] vs. 2142 [IQR, 2015–2198]; *p* = 0.869).

In contrast, POD3 LGI was significantly higher in the operative group (3029 [IQR, 2698–4666] vs. 2436 [IQR, 1911–3162]; *p* = 0.036). The absolute increase in LGI from POD1 to POD3 was also greater among patients requiring operative source control (1015 [IQR, 729–1518] vs. 462 [IQR, −259 to 1368]; *p* = 0.036). The POD3/POD1 LGI ratio was numerically higher in the operative group, but the difference was not statistically significant (1.46 [IQR, 1.35–1.59] vs. 1.23 [IQR, 0.88–1.49]; *p* = 0.096) (Table 3).

### 3.4. Discriminatory Performance of Postoperative Biomarkers

POD3 NLR demonstrated the highest observed discriminatory performance for subsequent operative source control, with an AUC of 0.901 (95% CI, 0.810–0.992; *p* < 0.001). At the data-derived cutoff of 12.6 selected using the maximum Youden index, POD3 NLR provided 95.8% sensitivity, 75.0% specificity, 82.1% positive predictive value, and 93.8% negative predictive value.

POD1 NLR also demonstrated good discriminatory performance, with an AUC of 0.834 (95% CI, 0.717–0.951; *p* < 0.001). At a cutoff of 14.2, sensitivity was 58.3%, whereas specificity and positive predictive value were both 100.0%; the negative predictive value was 66.7%. This pattern suggests that POD1 NLR had limited sensitivity but high specificity for subsequent operative source control.

POD1 CRP also demonstrated good discrimination, with an AUC of 0.798 (95% CI, 0.649–0.947; *p* < 0.001). A cutoff of 43.5 mg/L yielded 95.8% sensitivity, 70.0% specificity, 79.3% positive predictive value, and 93.3% negative predictive value. POD3 CRP had an AUC of 0.756 (95% CI, 0.604–0.908; *p* < 0.001); at a cutoff of 178 mg/L, sensitivity was 91.7% and specificity was 65.0%.

Both POD1 and POD3 CRP-to-albumin ratios showed significant discriminatory ability, with AUC values of 0.756 and 0.762, respectively. A POD3 CRP-to-albumin ratio cutoff of 6.85 provided 91.7% sensitivity, 65.0% specificity, 75.9% positive predictive value, and 86.7% negative predictive value.

POD3 WBC count had an AUC of 0.708 (95% CI, 0.556–0.861; *p* = 0.008), whereas POD1 WBC count did not demonstrate significant discrimination. Similarly, POD3 glucose had an AUC of 0.677 (95% CI, 0.516–0.838; *p* = 0.031), while POD1 glucose was not significant.

POD3 LGI showed modest discriminatory performance, with an AUC of 0.693 (95% CI, 0.533–0.852; *p* = 0.018). At a cutoff of 2593.5, POD3 LGI provided 87.5% sensitivity, 50.0% specificity, 67.7% positive predictive value, and 76.9% negative predictive value. POD1 LGI did not demonstrate significant discrimination.

In the subgroup without diabetes mellitus, POD3 LGI and ΔLGI each had an AUC of 0.709. A POD3 LGI cutoff of 2660 yielded 83.3% sensitivity and 58.8% specificity. A ΔLGI cutoff of 322 provided 94.4% sensitivity, 47.1% specificity, and an 88.9% negative predictive value. POD1 LGI and the POD3/POD1 LGI ratio were not statistically significant in this subgroup. Two-sided 95% confidence intervals for sensitivity, specificity, positive predictive value, and negative predictive value were calculated using the exact binomial Clopper–Pearson method (Table 4).

In the parsimonious Firth penalized logistic regression models adjusted for BMI and HIPEC agent, both principal biomarkers remained associated with subsequent operative source control. Each 10 mg/L increase in POD1 CRP was associated with higher adjusted odds of operative source control (adjusted odds ratio [aOR], 1.52; 95% CI, 1.10–2.56; *p* = 0.006). Using the corrected NLR values, each 5-unit increase in POD3 NLR was associated with an approximately fourfold increase in the adjusted odds of operative source control (aOR, 3.91; 95% CI, 1.57–20.23; *p* = 0.001). Complete model estimates, including those for BMI and HIPEC agent, are presented in Table 5. Given the limited cohort size and wide confidence intervals, these adjusted estimates were considered exploratory.

**Figure 1:**
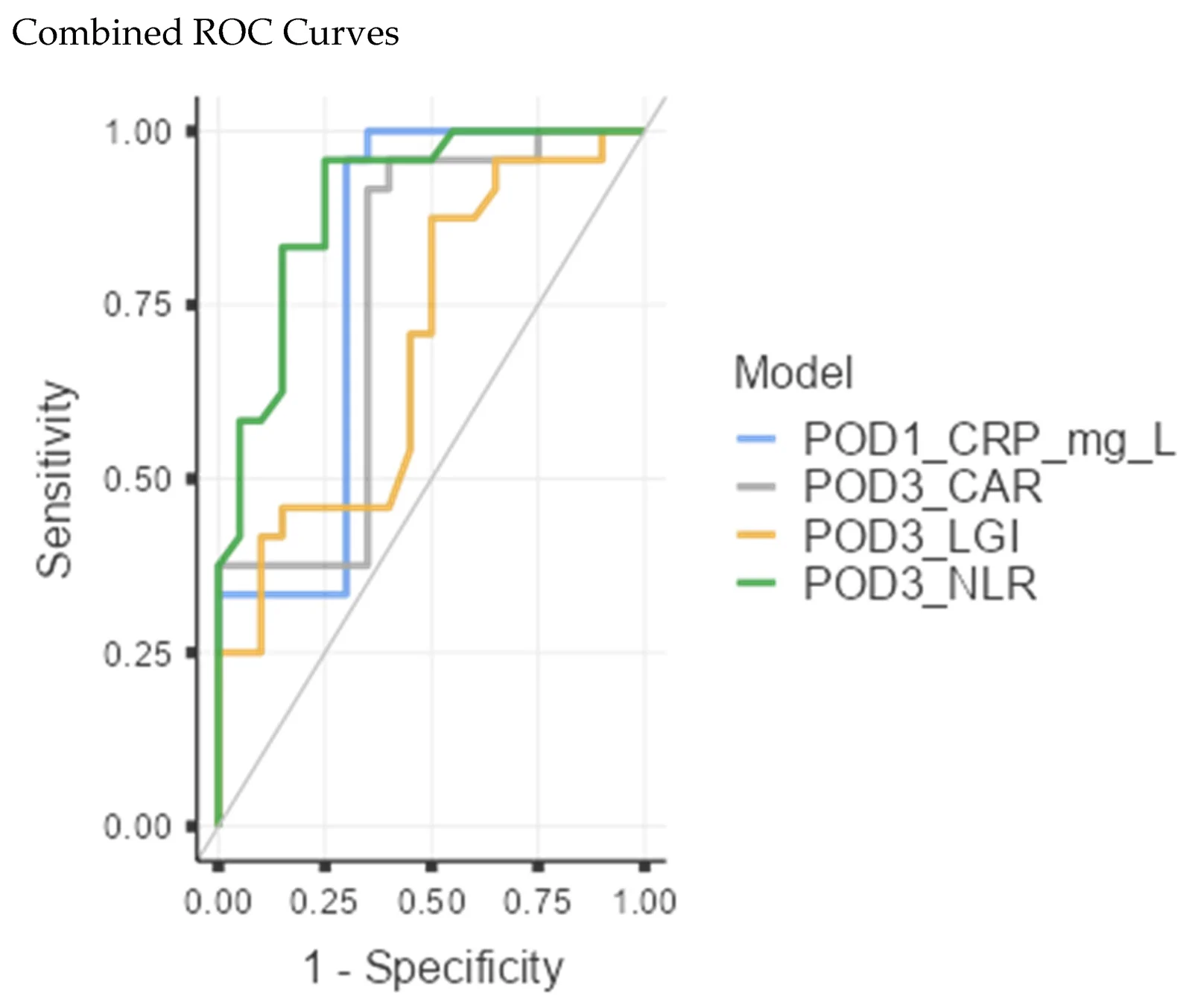
Receiver operating characteristic curves of selected postoperative biomarkers for discriminating between subsequent operative and non-operative management. Receiver operating characteristic curves demonstrating the discriminatory performance of postoperative day 1 C-reactive protein, postoperative day 3 neutrophil-to-lymphocyte ratio, postoperative day 3 C-reactive protein-to-albumin ratio, and postoperative day 3 leukoglycemic index for subsequent operative source control. POD3 NLR had the highest observed area under the curve.

The combined ROC curves for POD1 CRP, POD3 NLR, POD3 CRP-to-albumin ratio, and POD3 LGI demonstrated that POD3 NLR had the highest observed AUC among the selected biomarkers (AUC, 0.901; 95% CI, 0.810–0.992) (Figure 1). The ROC analyses were unadjusted, whereas the Firth penalized logistic regression models provided exploratory adjustment for BMI and HIPEC agent.

## 4. Discussion

The present study evaluated whether routinely available early postoperative inflammatory and metabolic biomarkers could discriminate, among patients who developed an anastomotic leak after CRS-HIPEC, those who subsequently required operative source control from those who were managed without reoperation through one month. POD3 NLR showed the highest observed discriminatory performance, with an AUC of 0.901 and a sensitivity and specificity of 95.8% and 75.0%, respectively, at a cutoff of 12.6. POD1 NLR also demonstrated good discrimination (AUC, 0.834), with high specificity but lower sensitivity at the selected cutoff. POD1 CRP retained good discriminatory performance, with an AUC of 0.798; a cutoff of 43.5 mg/L provided 95.8% sensitivity and a negative predictive value of 93.3%. In addition, the increase in NLR from POD1 to POD3 was significantly greater in patients who subsequently required operative source control. POD3 CRP-to-albumin ratio showed moderate discrimination, whereas POD3 LGI showed more limited performance, primarily because of its low specificity. These findings suggest that serial assessment of early postoperative biomarkers may provide supportive information for risk stratification but should not be regarded as a stand-alone indication for reoperation. Because the exact temporal relationship between biomarker sampling and non-operative leak-directed interventions could not be consistently reconstructed, the evaluated biomarkers should not be interpreted as predictors of failure after a standardized course of non-operative treatment. Nor can they be interpreted as general screening markers for anastomotic leakage among all patients undergoing CRS-HIPEC, because the study included only patients who were subsequently diagnosed with leakage. Rather, within this selected cohort, they represent early postoperative markers associated with the later need for operative source control.

The clinical question addressed in this study differs from that of most previous investigations. Earlier CRS-HIPEC studies primarily examined risk factors for the occurrence of gastrointestinal leakage or evaluated biomarkers for the detection of general infectious and major postoperative complications [2,3,4,6,7]. In contrast, the present analysis was restricted to patients who developed an anastomotic leak, and the outcome of interest was subsequent operative source control. This distinction is clinically relevant because selected stable patients with localized contamination may be treated with antimicrobial therapy, image-guided drainage, or endoscopic intervention, whereas uncontrolled contamination, generalized peritonitis, physiological deterioration, or failure of non-operative measures generally necessitates surgery [5]. Anastomotic leakage may also be recognized later after CRS-HIPEC than after conventional colorectal surgery, with reported median diagnostic times of 7 and 5 days, respectively [13]. Therefore, objective biomarker patterns that increase concern before overt clinical deterioration may help guide surveillance, imaging, and escalation of treatment.

Among the POD1 biomarkers, POD1 CRP demonstrated a clinically useful rule-out profile. Although POD1 NLR had a higher observed AUC than POD1 CRP (0.834 vs. 0.798), POD1 CRP provided substantially higher sensitivity and negative predictive value at the selected cutoff. Median POD1 CRP was 71 mg/L in patients who subsequently required operative source control, compared with 26.5 mg/L in those managed without reoperation. At a cutoff of 43.5 mg/L, POD1 CRP yielded 95.8% sensitivity and a negative predictive value of 93.3%. Within this selected cohort of patients who were subsequently diagnosed with anastomotic leakage, a lower POD1 CRP was associated with a lower probability of later operative source control. However, because patients without anastomotic leakage were not included, POD1 CRP cannot be interpreted as a general screening marker for anastomotic leakage or postoperative complications in the overall CRS-HIPEC population. The broader colorectal literature has consistently emphasized the rule-out value of postoperative CRP for anastomotic leakage [8]. A prospective colorectal cohort published in *Diagnostics* similarly demonstrated useful discriminatory performance of early postoperative serum CRP, particularly on POD3 and POD4 [10]. More recent evidence suggests that serial CRP trajectories may provide additional information beyond isolated absolute measurements [14]. Studies conducted specifically in CRS-HIPEC populations have also associated postoperative CRP concentrations and CRP kinetics with major or infectious complications [6,7,15,16]. However, these studies evaluated the occurrence of general postoperative morbidity rather than subsequent operative source control among patients with established anastomotic leakage. Furthermore, CRS-HIPEC itself induces a substantial systemic inflammatory response; therefore, thresholds derived from conventional colorectal surgery or unselected CRS-HIPEC cohorts cannot be directly extrapolated to the population examined in the present study.

POD3 NLR demonstrated the strongest observed discrimination. Median POD3 NLR was 21.0 in the operative group and 9.8 in the non-operative group. At a cutoff of 12.6, POD3 NLR provided 95.8% sensitivity, 75.0% specificity, 82.1% positive predictive value, and 93.8% negative predictive value. POD1 NLR also showed good discrimination, with an AUC of 0.834; however, at the selected cutoff of 14.2, it demonstrated high specificity (100.0%) but lower sensitivity (58.3%). Furthermore, the increase in NLR from POD1 to POD3 was significantly greater in patients who subsequently required operative source control, supporting the potential value of serial NLR assessment. NLR integrates neutrophil-predominant inflammatory activation and stress-associated relative lymphopenia and may therefore reflect the persistence and progression of systemic inflammation more effectively than either component alone. A prospective study of 204 patients undergoing stapled colorectal anastomosis found that POD3 NLR had an AUC of 0.74 and POD5 NLR an AUC of 0.93, whereas the corresponding CRP AUCs were 0.54 and 0.70 [11]. In another rectal cancer cohort, POD5 NLR predicted symptomatic leakage with an AUC of 0.818, with 76.5% sensitivity and 79.4% specificity at a cutoff of 6.54 [17]. A systematic review likewise reported an association between postoperative NLR and gastrointestinal anastomotic leakage, although measurement timing, patient populations, definitions of leakage, and proposed cutoff values varied considerably [18]. The higher cutoff observed in the present study compared with conventional colorectal surgery may reflect the substantial inflammatory burden associated with CRS-HIPEC, the restriction of the cohort to patients who had already developed an anastomotic leak, and the different outcome of subsequent operative source control rather than leakage occurrence itself. Nevertheless, because formal pairwise comparisons between AUCs were not performed, POD3 NLR should be described as having the highest observed AUC rather than being statistically superior to the other biomarkers.

The CRP-to-albumin ratio may theoretically provide broader physiological information by combining the acute inflammatory response with changes in albumin associated with vascular permeability, fluid balance, nutritional reserve, and postoperative stress. In the present cohort, both POD1 and POD3 CRP-to-albumin ratios were higher in patients requiring operative source control. The increase in the ratio between POD1 and POD3 was also greater in the operative group. POD3 CRP-to-albumin ratio had an AUC of 0.762, and a cutoff of 6.85 provided 91.7% sensitivity but only 65.0% specificity. Previous colorectal surgery research has suggested that the CRP-to-albumin ratio may be associated with anastomotic leakage and may potentially provide more information than CRP or albumin alone [19]. However, albumin levels did not differ between the groups in the present cohort, and the AUC of POD3 CRP-to-albumin ratio was similar to that of POD3 CRP. Consequently, the current findings do not demonstrate incremental discriminatory value of the ratio beyond CRP alone.

WBC count, serum glucose, and LGI generally showed weaker performance. POD1 WBC count, glucose, and LGI were comparable between the operative and non-operative groups and did not demonstrate significant discrimination. By POD3, however, all three parameters were higher among patients requiring operative source control. POD3 LGI had an AUC of 0.693, with 87.5% sensitivity but only 50.0% specificity at the selected cutoff. In patients without diabetes mellitus, POD3 LGI and the absolute change in LGI each had an AUC of 0.709. A change in LGI cutoff of 322 provided 94.4% sensitivity and a negative predictive value of 88.9%, but specificity remained only 47.1%. The persistence of the association after the exclusion of patients with diabetes suggests that the observed signal was not attributable exclusively to chronic dysglycemia. Nevertheless, the limited specificity indicates that LGI is unlikely to be clinically useful as an isolated decision threshold. Because published evidence regarding LGI has arisen predominantly from cardiovascular rather than gastrointestinal surgery [12], the present findings should be considered exploratory and hypothesis-generating.

Important differences in baseline and treatment-related characteristics were observed between the groups. Patients requiring operative source control had a higher median BMI than patients managed without reoperation. Neoadjuvant treatment was less frequent in the operative group, and the distribution of HIPEC agents differed significantly, with oxaliplatin administered more frequently among patients who underwent reoperation. These differences may reflect variation in primary tumor type, patient selection, institutional protocols, surgical complexity, or other unmeasured characteristics. They may also have influenced both postoperative biomarker responses and the clinical threshold for operative intervention. To address these imbalances, parsimonious Firth penalized logistic regression models were constructed for POD1 CRP and POD3 NLR, with adjustment for BMI and HIPEC agent. Both biomarker associations remained statistically significant after adjustment. However, neoadjuvant treatment could not be reliably incorporated because data were unavailable for nine patients, and the small sample size limited the number of covariates that could be included. Therefore, residual confounding remains possible, and the adjusted associations should be interpreted as exploratory rather than as evidence of independent or causal effects. Accordingly, the observed associations should not be interpreted as demonstrating that POD1 CRP or POD3 NLR reflects the biological severity of anastomotic leakage independently of BMI, HIPEC regimen, or other unmeasured factors. In particular, the higher frequency of oxaliplatin use in the operative group should not be interpreted as evidence that oxaliplatin directly caused more severe anastomotic leakage.

From a clinical perspective, the results support serial and contextual interpretation of postoperative biomarkers rather than reliance on a single numerical threshold. Within the selected population of patients who were subsequently diagnosed with anastomotic leakage, a lower POD1 CRP was associated with a relatively lower risk of later operative source control, whereas an elevated POD1 NLR provided early supportive evidence of higher risk. By POD3, persistent or increasing NLR may raise further concern regarding an inadequately controlled inflammatory process. The significantly greater increase in NLR, together with the greater increases in CRP-to-albumin ratio, WBC count, and LGI between POD1 and POD3 among operative patients, suggests that evolving biomarker trajectories may provide additional information beyond isolated measurements. However, laboratory biomarkers cannot determine whether intra-abdominal contamination has been anatomically controlled. They must therefore be interpreted together with hemodynamic status, organ dysfunction, physical examination findings, drain characteristics, radiological findings, and response to initial treatment. The proposed thresholds should be viewed as potential aids for surveillance, imaging, and risk stratification rather than automatic indications for reoperation. Combining POD1 CRP and POD3 NLR may potentially improve discrimination by integrating complementary early and evolving inflammatory information. However, the limited sample size and the lack of a prespecified modeling framework precluded the development and validation of a reliable multivariable risk stratification model in the present study. Similarly, SOFA, qSOFA, and other sepsis-related severity scores could not be evaluated because the required patient-level physiological and organ dysfunction variables were not consistently available in the pooled retrospective dataset. Future prospective studies should assess whether combining biomarkers with standardized clinical severity scores provides incremental value beyond individual biomarkers alone.

Several features strengthen the present study. It addresses an uncommon but clinically important complication of CRS-HIPEC and focuses on a management question that has received limited attention: whether operative source control will be required after an anastomotic leak has developed. All POD1 and POD3 laboratory measurements in patients who subsequently underwent reoperation were obtained before operative source control, ensuring that these values did not reflect the biological response to reoperation. Assessment of both POD1 and POD3 measurements allowed evaluation of very early biomarker levels and short-term postoperative changes. The simultaneous analysis of CRP, NLR, CRP-to-albumin ratio, WBC count, glucose, and LGI provided information across inflammatory, nutritional, and metabolic domains. In addition, the analysis restricted to patients without diabetes mellitus explored whether LGI findings were driven primarily by pre-existing dysglycemia.

Several limitations should be considered. First, the retrospective design introduces the possibility of selection bias, incomplete documentation, and residual confounding. Second, the cohort included only 44 patients, of whom 24 required operative source control, resulting in limited precision and relatively wide confidence intervals for several ROC estimates. Third, four patients who underwent reoperation before completion of the POD3 laboratory assessment were excluded, including two patients reoperated for anastomotic leakage and two reoperated for postoperative bleeding. Although this restriction prevented operative intervention from influencing subsequent biomarker values, it introduced landmark-related selection bias by limiting the analysis to patients who remained free from reoperation through POD3. A separate POD1 analysis of the patients undergoing early reoperation for anastomotic leakage was not performed because only two such patients were available, precluding meaningful statistical estimation. The findings are therefore not applicable to patients requiring very early surgical intervention. Fourth, the decision to perform operative source control was not based on a standardized study protocol and may have varied according to surgeon judgment, institutional practice, imaging interpretation, patient condition, and the availability of interventional radiology or endoscopic treatment. Clinicians were also aware of routine laboratory findings, introducing the possibility that the evaluated biomarkers influenced the management outcome they were intended to discriminate. Fifth, although institution-level patient and operative source control counts were available descriptively, the small number of patients within each institution and the non-uniform institutional origin of the private-hospital cases prevented reliable adjustment for center-level clustering or between-center differences in treatment strategies. Neoadjuvant treatment data were incomplete and were analyzed using available cases. Multiple biomarkers and time points were examined without correction for multiple comparisons, increasing the possibility of chance findings. Furthermore, because POD1 CRP and POD3 NLR were selected for adjusted modeling after their observed discriminatory performance had been examined rather than according to a fully prespecified analysis plan, these data-informed, post hoc models are susceptible to selection-related optimism and overfitting, and the statistical significance of the adjusted associations should therefore be interpreted cautiously. Standardized patient-level indications for reoperation and detailed intraoperative findings were not consistently available in the pooled anonymized dataset. Therefore, complete retrospective ISREC grading could not be reliably assigned to the entire cohort. Although the timing of leakage diagnosis was available in predefined postoperative day categories, the exact patient-level day of diagnosis and the interval between diagnosis and operative source control were not consistently available in the pooled dataset. Therefore, the median postoperative day of leakage diagnosis and the median diagnosis-to-reoperation interval could not be reliably calculated. The exact timing of imaging, antimicrobial treatment, and radiological or endoscopic interventions relative to POD1 and POD3 biomarker sampling was also not consistently available. Finally, the cutoff values were derived and evaluated in the same small cohort and may therefore overestimate discriminatory performance. Because the ROC-derived cutoffs and their operating characteristics were selected and evaluated in the same small cohort without internal resampling or external validation, the reported AUCs, sensitivities, specificities, and predictive values are likely subject to optimistic bias and overfitting. Positive and negative predictive values were influenced by the 54.5% prevalence of operative source control in this selected population and may differ substantially in other settings. No formal pairwise comparison of AUCs, internal resampling validation, or external validation was performed. Detailed patient-level indications for reoperation, including generalized peritonitis, hemodynamic deterioration, uncontrolled collections, or failure of drainage, as well as the exact distribution of individual operative procedures, were not consistently available in the pooled retrospective dataset. The results should therefore be considered exploratory and should not be generalized to all patients undergoing CRS-HIPEC, patients requiring intervention before POD3, or patients undergoing conventional gastrointestinal surgery. Larger prospective multicenter studies with standardized definitions, management criteria, predefined primary biomarkers, and external validation are required before the proposed thresholds can be incorporated into routine clinical practice.

## 5. Conclusions

Within this selected retrospective cohort of patients who developed anastomotic leakage after CRS-HIPEC, POD1 CRP, POD1 NLR, and POD3 NLR were associated with and showed apparent discrimination for subsequent operative source control. POD1 CRP provided high sensitivity and negative predictive value, whereas POD1 NLR showed high specificity at the selected cutoff. POD3 NLR had the highest observed AUC and provided high sensitivity and negative predictive value; the greater increase in NLR from POD1 to POD3 among patients requiring surgery further supports the potential value of serial assessment. CRP-to-albumin ratio and LGI may provide complementary information, although their additional clinical value remains uncertain. These exploratory findings require confirmation in larger prospective multicenter studies with standardized management criteria and external validation before the proposed thresholds can be incorporated into routine clinical practice.

## Figures and Tables

**Table 1:** Baseline demographic, clinical, and operative characteristics according to subsequent management strategy.

Characteristic	Overall (*n* = 44)	Non-Operative (*n* = 20)	Operative (*n* = 24)	*p* Value
**Demographic and clinical characteristics**				
Age, years	65 [54–68.2]	62 [54–65.8]	65 [54.2–69]	0.492
Female sex, *n* (%)	27 (61.4)	12 (60.0)	15 (62.5)	1.000
BMI, kg/m^2^	26 [24–32.2]	24 [20–26]	29 [26–35.1]	**<0.001**
Diabetes mellitus, *n* (%)	9 (20.5)	3 (15.0)	6 (25.0)	0.477
Neoadjuvant treatment, *n*/available (%)	17/35 (48.6)	12/17 (70.6)	5/18 (27.8)	**0.018**
PCI score	14 [9–18]	15 [4–25]	13.5 [9–17.2]	0.953
CC score 1, *n* (%)	11 (25.0)	4 (20.0)	7 (29.2)	0.728
**Operative characteristics**				
Operative duration, min	300 [200–302.5]	290 [180–305]	300 [200–302.5]	0.269
HIPEC agent, *n* (%)				**0.002**
Oxaliplatin	24 (54.5)	6 (30.0)	18 (75.0)	
Mitomycin C	16 (36.4)	10 (50.0)	6 (25.0)	
Cisplatin	4 (9.1)	4 (20.0)	0 (0.0)	
HIPEC duration, min	60 [60–60]	60 [60–60]	60 [60–90]	0.370
Anastomosis type, *n* (%)				0.095
Colocolic	16 (36.4)	7 (35.0)	9 (37.5)	
Enterocolic	15 (34.1)	10 (50.0)	5 (20.8)	
Colorectal	9 (20.5)	3 (15.0)	6 (25.0)	
Enterorectal	4 (9.1)	0 (0.0)	4 (16.7)	

Data are presented as median [interquartile range] or *n* (%), unless otherwise indicated. Percentages for neoadjuvant treatment were calculated using available cases (overall *n* = 35; non-operative *n* = 17; operative *n* = 18). Continuous variables were compared using the Mann–Whitney U test; binary categorical variables using Fisher’s exact test; and multilevel categorical variables using the Fisher–Freeman–Halton exact test with Monte Carlo simulation (300,000 resamples). Protective stoma was not tabulated because all patients were recorded as having no protective stoma. Abbreviations: BMI, body mass index; PCI, peritoneal cancer index; CC, completeness of cytoreduction; HIPEC, hyperthermic intraperitoneal chemotherapy.

**Table 2:** Postoperative day 1 and day 3 inflammatory and metabolic parameters according to management strategy.

Parameter	Overall (*n* = 44)	Non-Operative (*n* = 20)	Operative (*n* = 24)	*p* Value
**Postoperative day 1**				
CRP, mg/L	59 [30.8–90]	26.5 [18–89]	71 [57.8–127.5]	<0.001
Albumin, g/L	29 [21–30.2]	29 [21.8–32]	29.5 [21–30]	0.592
CRP/albumin ratio	2.07 [1.43–3]	1.24 [0.65–2.78]	2.26 [1.8–6.07]	0.001
NLR	12.7 [9.6–15.1]	9.8 [7.0–12.7]	14.9 [12.4–16.7]	<0.001
WBC, ×10^3^/uL	14 [13–14.2]	14 [13–14]	14 [13–15]	0.699
Glucose, mg/dL	158.5 [147.5–208.8]	156.5 [150.8–177.2]	172.5 [146–214]	0.430
Leukoglycemic index	2184 [2043–3082]	2177 [2054–2438]	2194 [2043–3109]	0.646
**Postoperative day 3**				
CRP, mg/L	197 [169.2–251.8]	170 [52.5–245.5]	203 [193.8–313]	0.004
Albumin, g/L	24 [23.8–25.8]	24 [24–25]	25.4 [20.4–25.8]	0.895
CRP/albumin ratio	7.52 [5.31–10.61]	5.31 [2.19–10.23]	7.92 [7.25–15.57]	0.003
NLR	14.9 [9.9–21.9]	9.8 [8.5–12.7]	21.0 [15.6–23.3]	<0.001
WBC, ×10^3^/uL	17 [14–17]	14.5 [13–17]	17 [16–17.2]	0.016
Glucose, mg/dL	183 [155.2–263.5]	175 [146.8–206.5]	209 [166.5–273.2]	0.046
Leukoglycemic index	3056 [2427–4480]	2672 [1930–3321]	3060 [2800–4722]	0.030
**Change from postoperative day 1 to day 3**				
Change in CRP, mg/L	133.5 [80–170.2]	80 [31.8–196.5]	140.5 [130–163.8]	0.101
Change in albumin, g/L	−4 [−6–2]	−1 [−9–2.9]	−4.2 [−6–−3]	0.338
Change in CRP/albumin ratio	5.65 [2.31–8.81]	2.31 [1.53–8.78]	6 [5.42–9.57]	0.014
Change in NLR	2.9 [0.3–6.8]	1.0 [−2.7–4.1]	4.8 [2.4–7.4]	0.009
Change in WBC, ×10^3^/uL	2 [0.8–4]	0.5 [−1–3.2]	2.5 [2–4]	0.019
Change in glucose, mg/dL	22.5 [−1.2–47.2]	8 [−8–41.2]	36.5 [17–48.8]	0.071
Change in leukoglycemic index	871 [222–1485]	381 [−198–1371]	1015 [763–1584]	0.022
POD3/POD1 leukoglycemic index ratio	1.41 [1.11–1.51]	1.19 [0.91–1.48]	1.44 [1.32–1.58]	0.061

All biomarker analyses included 44 patients (20 non-operative and 24 operative), with no missing POD1 or POD3 laboratory values. Data are presented as median [interquartile range]. Between-group comparisons were performed using the Mann–Whitney U test. Change values were calculated as postoperative day 3 minus postoperative day 1. The leukoglycemic index was calculated as WBC (×10^3^/uL) × glucose (mg/dL). Patients who underwent reoperation before postoperative day 3 were excluded. Abbreviations: CRP, C-reactive protein; NLR, neutrophil-to-lymphocyte ratio; WBC, white blood cell count; POD, postoperative day.

**Table 3:** Leukoglycemic index parameters in patients without diabetes mellitus according to subsequent management strategy.

LGI Parameter	Non-Operative (*n* = 17)	Operative (*n* = 18)	*p* Value
POD1 leukoglycemic index	2142 [2015–2198]	2074 [2032–3056]	0.869
POD3 leukoglycemic index	2436 [1911–3162]	3029 [2698–4666]	0.036
Change in leukoglycemic index (POD3-POD1)	462 [−259–1368]	1015 [729–1518]	0.036
POD3/POD1 leukoglycemic index ratio	1.23 [0.88–1.49]	1.46 [1.35–1.59]	0.096

Data are presented as median [interquartile range]. Patients with diabetes mellitus were excluded from this analysis. Between-group comparisons were performed using the Mann–Whitney U test. The leukoglycemic index was calculated as WBC (×10^3^/microliter) × glucose (mg/dL). Abbreviations: LGI, leukoglycemic index; POD, postoperative day; WBC, white blood cell count.

**Table 4:** Discriminatory performance of postoperative biomarkers for subsequent operative source control.

**Panel A.** **Overall Cohort (** ***n*** ** = 44)**								
**Parameter**	**AUC**	**95% CI**	***p*** **Value**	**Cutoff**	**Sensitivity, % (95% CI)**	**Specificity, % (95% CI)**	**PPV, % (95% CI)**	**NPV, % (95% CI)**
POD1 CRP, mg/L	0.798	0.649–0.947	<0.001	≥43.5	95.8 (78.9–99.9)	70.0 (45.7–88.1)	79.3 (60.3–92.0)	93.3 (68.1–99.8)
POD3 CRP, mg/L	0.756	0.604–0.908	<0.001	≥178	91.7 (73.0–99.0)	65.0 (40.8–84.6)	75.9 (56.5–89.7)	86.7 (59.5–98.3)
POD1 CRP/albumin ratio	0.756	0.607–0.906	<0.001	≥1.47	95.8 (78.9–99.9)	55.0 (31.5–76.9)	71.9 (53.3–86.3)	91.7 (61.5–99.8)
POD3 CRP/albumin ratio	0.762	0.610–0.915	<0.001	≥6.85	91.7 (73.0–99.0)	65.0 (40.8–84.6)	75.9 (56.5–89.7)	86.7 (59.5–98.3)
POD1 NLR	0.834	0.717–0.951	<0.001	≥14.2	58.3 (36.6–77.9)	100.0 (83.2–100.0)	100.0 (76.8–100.0)	66.7 (47.2–82.7)
POD3 NLR	0.901	0.810–0.992	<0.001	≥12.6	95.8 (78.9–99.9)	75.0 (50.9–91.3)	82.1 (63.1–93.9)	93.8 (69.8–99.8)
POD1 WBC, ×10^3^/µL	0.533	0.368–0.699	0.693	≥15	33.3 (15.6–55.3)	85.0 (62.1–96.8)	72.7 (39.0–94.0)	51.5 (33.5–69.2)
POD3 WBC, ×10^3^/µL	0.708	0.556–0.861	0.008	≥16	79.2 (57.8–92.9)	55.0 (31.5–76.9)	67.9 (47.6–84.1)	68.8 (41.3–89.0)
POD1 glucose, mg/dL	0.571	0.395–0.747	0.431	≥170	58.3 (36.6–77.9)	75.0 (50.9–91.3)	73.7 (48.8–90.9)	60.0 (38.7–78.9)
POD3 glucose, mg/dL	0.677	0.516–0.838	0.031	≥201	58.3 (36.6–77.9)	75.0 (50.9–91.3)	73.7 (48.8–90.9)	60.0 (38.7–78.9)
POD1 leukoglycemic index	0.542	0.366–0.718	0.642	≥3016	45.8 (25.6–67.2)	75.0 (50.9–91.3)	68.8 (41.3–89.0)	53.6 (33.9–72.5)
POD3 leukoglycemic index	0.693	0.533–0.852	0.018	≥2593.5	87.5 (67.6–97.3)	50.0 (27.2–72.8)	67.7 (48.6–83.3)	76.9 (46.2–95.0)
**Panel B. Patients Without Diabetes Mellitus (** ***n*** ** = 35)**								
**Parameter**	**AUC**	**95% CI**	***p*** **Value**	**Cutoff**	**Sensitivity, % (95% CI)**	**Specificity, % (95% CI)**	**PPV, % (95% CI)**	**NPV, % (95% CI)**
POD1 leukoglycemic index	0.518	0.316–0.720	0.861	≥3045	33.3 (13.3–59.0)	88.2 (63.6–98.5)	75.0 (34.9–96.8)	55.6 (35.3–74.5)
POD3 leukoglycemic index	0.709	0.533–0.886	0.020	≥2660	83.3 (58.6–96.4)	58.8 (32.9–81.6)	68.2 (45.1–86.1)	76.9 (46.2–95.0)
Δ leukoglycemic index	0.709	0.532–0.886	0.020	≥322	94.4 (72.7–99.9)	47.1 (23.0–72.2)	65.4 (44.3–82.8)	88.9 (51.8–99.7)
POD3/POD1 LGI ratio	0.667	0.477–0.856	0.084	≥1.16	94.4 (72.7–99.9)	47.1 (23.0–72.2)	65.4 (44.3–82.8)	88.9 (51.8–99.7)

AUC and 95% confidence intervals were calculated using the DeLong method. The *p* value tests AUC against 0.50. Cutoffs were selected using the maximum Youden index. The 95% confidence intervals for sensitivity, specificity, PPV, and NPV were calculated using the exact binomial Clopper–Pearson method. The data-derived cutoffs are exploratory and should not be interpreted as validated clinical decision thresholds. LGI was calculated as WBC (×10^3^/µL) × glucose (mg/dL). ΔLGI = POD3 LGI-POD1 LGI. Panel B includes 17 non-operative and 18 operative patients without diabetes mellitus. All POD3 measurements were obtained before operative source control. Abbreviations: AUC, area under the curve; CI, confidence interval; CRP, C-reactive protein; LGI, leukoglycemic index; NLR, neutrophil-to-lymphocyte ratio; NPV, negative predictive value; POD, postoperative day; PPV, positive predictive value; WBC, white blood cell count.

**Table 5:** Firth penalized logistic regression models for operative source control.

Model and Covariate	Coefficient	Adjusted OR	95% CI	*p* Value
**Model 1: POD1 CRP**				
POD1 CRP, per 10 mg/L	0.422	1.52	1.10–2.56	0.006
BMI, per 1 kg/m^2^	0.370	1.45	1.15–2.12	<0.001
Mitomycin C	-	Reference	-	-
Oxaliplatin vs. mitomycin C	3.317	27.57	2.96–793.32	0.002
Cisplatin vs. mitomycin C	−1.422	0.24	0.001–10.21	0.450
**Model 2: POD3 NLR**				
POD3 NLR, per 5 units	1.362	3.91	1.57–20.23	0.001
BMI, per 1 kg/m^2^	0.373	1.45	1.13–2.32	0.001
Mitomycin C	-	Reference	-	-
Oxaliplatin vs. mitomycin C	3.052	21.17	1.94–1605.87	0.009
Cisplatin vs. mitomycin C	−0.437	0.65	0.003–32.57	0.831

The outcome event was operative source control; non-operative management was the reference outcome. Each model included 44 patients and 24 events. Mitomycin C was the reference category for HIPEC agent. Adjusted odds ratios are reported with profile-likelihood 95% confidence intervals and penalized likelihood-ratio *p* values. HIPEC agent estimates were included for covariate adjustment and should not be interpreted as causal treatment effects. The cisplatin category contained no operative events, resulting in imprecise estimates despite Firth penalization. Abbreviations: BMI, body mass index; CI, confidence interval; CRP, C-reactive protein; HIPEC, hyperthermic intraperitoneal chemotherapy; NLR, neutrophil-to-lymphocyte ratio; OR, odds ratio; POD, postoperative day.

## Data Availability

The data supporting the findings of this study are available from the corresponding author upon reasonable request. The data are not publicly available because they contain potentially identifiable clinical information and are subject to patient-confidentiality, institutional, and ethical restrictions.
